# 3-(1H-Benzo[*d*]imidazol-6-yl)-5-(4-fluorophenyl)-1,2,4-oxadiazole (DDO7232), a Novel Potent Nrf2/ARE Inducer, Ameliorates DSS-Induced Murine Colitis and Protects NCM460 Cells against Oxidative Stress via ERK1/2 Phosphorylation

**DOI:** 10.1155/2018/3271617

**Published:** 2018-05-20

**Authors:** Li-Li Xu, Tian Liu, Lei Wang, Li Li, Yu-Feng Wu, Cui-Cui Li, Bin Di, Qi-Dong You, Zheng-Yu Jiang

**Affiliations:** ^1^State Key Laboratory of Natural Medicines and Jiangsu Key Laboratory of Drug Design and Optimization, China Pharmaceutical University, Nanjing 210009, China; ^2^Department of Medicinal Chemistry, School of Pharmacy, China Pharmaceutical University, Nanjing 210009, China; ^3^Key Laboratory on Protein Chemistry and Structural Biology and Key Laboratory of Drug Quality Control and Pharmacovigilance, Ministry of Education, China Pharmaceutical University, Nanjing 210009, China

## Abstract

Ulcerative colitis (UC) is a common inflammatory bowel disease that can destroy the integrity of the colon and increase the risk of colorectal cancer. Oxidative stress is one of the critical pathogenic factors for UC, further impairing the entire affected colon. The Nrf2-ARE signaling pathway plays an important role in counteracting oxidative and electrophilic stress. Activation of the Nrf2-ARE pathway provides an indispensable defense mechanism for the treatment of UC. In this study, we identified a novel effective Nrf2 activator, DDO7232, which showed protective effects on NCM460 cells and therapeutic effects on DSS-induced colitis in mice. Mechanistic studies indicated that the Nrf2-ARE-inducing activity of DDO7232 was based on the activation of the ERK1/2 phosphorylation. The phosphorylation of Nrf2 Ser40 by p-ERK triggered the transport of Nrf2 into the nucleus and drove the expression of Nrf2-dependent antioxidant proteins. These results not only revealed the antioxidant mechanisms of DDO7232 but also provided an effective therapeutic option for the treatment of UC.

## 1. Introduction

Ulcerative colitis (UC) is a chronic relapsing-remitting inflammatory disease that has received widespread attention and research [1]. The exact etiology of UC remains unclear, although there is some evidence that contributes to understanding the pathogenesis of the disease [2, 3].

UC increases the risk of colorectal cancer, one of the most common malignancies in human beings [4, 5]. Considerable efforts have been devoted to the development of medicinal therapies, including antibiotics, steroids, immunomodulators, and biologics [6]. However, most current therapeutic approaches rely on immunosuppressive methods, resulting in nonspecific mechanisms and potential side effects [7].

Identifying the etiology of UC is important for improving the current therapeutic strategies and preventing colorectal cancer [8, 9]. There are plenty of pathogenic factors associated with inflammation. Among them, oxidative stress is one of the major factors in the transformation of chronic inflammation into cancer and other diseases. The deficiency of antioxidant/detoxification enzymes, along with increases in reactive oxygen species (ROS) and nitrogen fragments, is harmful to colonic homeostasis [10]. Nuclear factor erythroid 2 p45-related factor 2 (Nrf2), a cytoprotective transcription factor [11, 12], regulates the expression of the downstream antioxidant/detoxification enzymes [13, 14]. During the early phase of inflammation-mediated tissue injury, the activation of Nrf2 might inhibit the production or expression of proinflammatory mediators including cytokines, chemokines, and cell adhesion molecules [10].

Under normal conditions, the formation and degradation of Nrf2 are in equilibrium [15]. Under stress conditions, the degradation of Nrf2 is inhibited. The newly synthesized Nrf2 accumulates and translocates into the nucleus to bind with the antioxidant responsive elements (AREs), inducing the transcription and protective functions of antioxidant genes [16]. In addition, Nrf2 can be modulated by multiple protein kinases, making it a promising regulatory node in cellular defense systems [17]. Nrf2 is a novel substrate of phosphorylated extracellular signal-regulated kinase (p-ERK) [18]. The phosphorylation of the Nrf2 Ser40 by p-ERK promotes the transport of Nrf2 into the nucleus and its binding to ARE, leading to the coordinated expression of antioxidant proteins including NAD(P)H/quinone oxidoreductase 1 (NQO1), heme oxygenase-1 (HO-1), glutamate-cysteine ligase modifier subunit (GCLM), glutamate-cysteine ligase catalytic subunit (GCLC), and several members of the glutathione S-transferase family [19, 20].

As a key node in protection against intense oxidative stress, Nrf2 regulates several antioxidant-signaling pathways in the colonic epithelium, making the activation of Nrf2 an alternative treatment option for patients with colitis [21, 22]. This was validated by a study which showed that Nrf2-deficient mice were more susceptible to dextran sulfate sodium- (DSS-) induced colitis [23]. The increased severity of colitis in Nrf2-deficient (Nrf2 (−/−)) mice is associated with the downregulation of phase II antioxidant/detoxifying enzymes. In the colon tissues of Nrf2 (−/−) mice, proinflammatory mediators/cytokines, such as interleukin 1*β* (IL-1*β*), interleukin 6 (IL-6), and tumor necrosis factor *α* (TNF-*α*), were significantly upregulated compared with the colon tissues of wild-type (Nrf2 (+/+)) mice [22, 24]. Sulforaphane, an Nrf2 activator, relieves DSS-induced colitis in C57BL/6 mice [25].

DDO7232, a novel Nrf2 activator, exhibits potent antioxidant and preliminary anti-inflammatory activity [26]. Here, we identified the protective mechanism of DDO7232 against DSS-induced oxidative damage in NCM460 colonic cells. DDO7232 downregulated the content of inflammatory factors in the culture medium of DSS-treated NCM460 cells through activation of the Nrf2-ARE signaling pathway. Mechanistic studies in NCM460 cells demonstrated that the activation of Nrf2-ARE by DDO7232 was mediated by the activation of ERK1/2 phosphorylation. Nrf2 Ser40, phosphorylated by p-ERK, facilitated the transport of Nrf2 into the nucleus, the transcription of Nrf2-dependent genes, and the expression of antioxidant proteins, resulting in a coordinated downregulation of ROS in NCM460 cells. Apart from the cytoprotective effects of DDO7232, in vivo research results clearly demonstrated its therapeutic effects on the DSS-induced mouse model of chronic ulcerative colitis by the activation of Nrf2-ARE pathway.

## 2. Materials and Methods

### 2.1. Cell Culture Conditions

Human NCM460 colonocytes (INCELL, San Antonio, TX) were cultured in Dulbecco's modified Eagle medium (Life Technology™, 1645798) supplemented with 10% (*v*/*v*) fetal bovine serum (FBS) (Gibco, NY, USA) and penicillin/streptomycin, in a humidified atmosphere of 5% CO_2_ and 95% air at 37°C. HepG2 cells, stably transfected with the ARE-luciferase reporter (HepG2-ARE-C8), were kindly provided by Professor Dr. A. N. Tony Kong (Rutgers University, Piscataway, NJ). The cells were cultured in modified RPMI-1640 medium (Gibco, NY, USA) with 10% FBS (Gibco, NY, USA) in a humidified atmosphere of 5% CO_2_ and 95% air at 37°C.

### 2.2. Compound Preparation

DDO7232 was synthesized as described previously [26]. A 10 mmol stock solution of DDO7232 in DMSO was stored at −20°C. Fresh cell culture medium was used to dilute the stock solutions.

### 2.3. ARE-Luciferase Activity Assay

HepG2-ARE-C8 cells were plated in 48-well plates (8 × 10^4^ cells/well) and incubated overnight. The cells were exposed to DDO7232 (20 *μ*M), with tBHQ serving as a positive control, DMSO as a negative control, and the luciferase cell culture lysis reagent as a blank. The cells were cotreated with inhibitors of MEK1/2 (PD098059, 20 *μ*M), PI3K (LY294002, 10 *μ*M), or P38 (SB203580, 5 *μ*M) (Beyotime, China). After 12 h of treatment, the medium was removed and 400 *μ*L of cold PBS was added to each well. Then, the cells were harvested using the luciferase cell culture lysis reagent [27]. After centrifugation, 20 *μ*L of the supernatant from the lysate was used for determining luciferase activity according to the manufacturer's protocol (Promega, Madison, WI). The luciferase activity was measured by using Luminoskan Ascent (Thermo Scientific, USA). The data were obtained in triplicate and expressed as a fold induction over the control.

Inductivity = (RLU_test_ − RLU_blank_)/(RLU_control_ − RLU_blank_), where RLU = relative light unit.

### 2.4. MTT Analysis

Cytotoxicity was determined by using the MTT assay. MTT was purchased from Sigma (St. Louis, MO). It was dissolved in phosphate-buffered saline (PBS) to a stock solution concentration of 5 mg/mL and stored at −20°C. After the cells were treated with a density gradient of test compounds or DMSO for 24 h, 20.0 *μ*L of MTT solution (5 mg/mL) was added to each well of 96-well plates and incubated for 4 h. Then, the solution was removed and 150.0 *μ*L of DMSO was added into each well to dissolve the water-soluble MTT-formazan crystals. The absorbance values (OD value) were recorded at 570 nm by an ELx800 absorbance microplate reader (BioTek, Vermont, USA).

(1) $$\begin{array}{c} {IC}_{50}=\left[\frac{1-\left({OD}_{test}-{OD}_{blank}\right)}{\left({OD}_{control}-{OD}_{blank}\right)}\right]\times 100\%. \end{array}$$

### 2.5. Western Blot

Anti-NQO1 (sc-271116) (1 : 500) monoclonal antibody was bought from Santa Cruz Biotechnology (Santa Cruz, CA, USA). Anti-*β*-actin (AP0060) (1 : 1000) and anti-Nrf2 (BS1258, 1 : 1000) monoclonal antibodies were purchased from Bioworld (Bioworld, USA). Rabbit polyclonal phospho-Nrf2 (Ser40) (ab76026) (1 : 1000) antibody was obtained from Abcam (Cambridge, UK). Antibody against p-ERK1/2 (#4370) (1 : 1000) was obtained from Cell Signaling Technology (Beverly, MA, USA). Anti-HO-1 (number 5853S) (1 : 1000) was purchased from Cell Signaling Technology (USA). Anti-GAPDH (number 60004-1-1g) (1 : 1000) mouse polyclonal and anti-histone H3 rabbit polyclonal antibodies (number 17168–1-AP) (1 : 1000) which were used as internal reference were purchased from Proteintech. The cells were washed once with ice-cold PBS and driven down with 1 mL of 1x pancreatic enzyme. The cells were centrifuged at 2500 rpm and resuspended and incubated for 1 h in 80 *μ*L of lysis buffer (50 mM Tris-HCl, 150 *μ*M NaCl, NP-40, 1 *μ*M EDTA, PMSF, NaF, Leu, and DTT). Then, the cells were centrifuged again at 12,000 rpm for 20 min at 4°C. The supernatant was retained, and the protein concentration was determined using the BCA assay with Varioskan flash (Thermo, Waltham, MA) at 562 nm. Samples were stored at −80°C until used.

A nuclear-cytosol extraction kit (KeyGEN, NJ, China) was used to isolate the nuclear and cytosol protein according to the manufacturer's instructions. The collected protein was stored at −80°C until use.

Equal amounts of total protein extract were separated by SDS-PAGE and then transferred onto PVDF membranes (PerkinElmer, Norwalk, CT, USA). After blocking with 1% BSA for 2 h, the membranes were incubated with the primary antibodies for 1 h at 37°C and then overnight at 4°C. After that, they were reacted with a DyLight 800-labeled secondary antibody (1 : 10,000) at 37°C for 1 h. The membranes were scanned through the Odyssey Infrared Imaging System (LI-COR, Lincoln, Nebraska, USA).

### 2.6. qRT-PCR

Total RNA from NCM460 cells was isolated using TRIzol (Invitrogen). The quantification and purity of the RNA samples were assessed by A260/A280 absorption, and RNA samples with ratios above 1.8 were stored at −80°C for further analysis. The RNA was reverse transcribed using a PrimeScript™ RT reagent kit as per the manufacturer's instructions. The following primer sequences were used: Nrf2 sense primer 5′-AACCACCCTGAAAGCACGC-3′ and antisense primer 5′-TGAAATGCCGGAGTCAGAATC-3′, HO-1 sense primer 5′-ATGGCCTCCCTGTACCACATC-3′ and antisense primer 5′-TGTTGCGCTCAATCTCCTCCT-3′, NQO1 sense primer 5′-CGCAGACCTTGTGATATTCCAG-3′ and antisense primer 5′-CGTTTCTTCCATCCTTCCAGG-3′, GCLM sense primer 5′-TTGGAGTTGCACAGCTGGATTC-3′ and antisense primer 5′-TGGTTTTACCTGTGCCCACTG-3′, GCLC sense primer 5′-GTCCTCAGGTGACATTCCAAGC-3′ and antisense primer 5′-TGTTCTTCAGGGGCTCCAGTC-3′, GAPDH sense primer 5′-AGGTCGGTGTGAACGGATTTG-3′ and antisense primer 5′-TGTAGACCATGTAGTTGAGGTCA-3′, ERK1 sense primer 5′-CGCTACACGCAGTTGCAGTACA-3′ and antisense primer 5′-AAGCGCAGCAGGATCTGGA-3′, and ERK2 sense primer 5′-TGTTCCCAAATGCTGACTCCAA-3′ and antisense primer 5′-TCGGGTCGTAATACTGCTCCAGATA-3′. Quantitative real-time RT-PCR analysis of Nrf2, NQO1, HO-1, GCLC, GCLM, and GAPDH was performed by using a StepOne Fast Real-Time PCR system (Applied Biosystems). The values given are relative to the fold change in the expression of the controls. Each cycle included denaturation at 95°C for 5 sec and combined annealing and extension at 60°C for 30 sec. A total of 40 cycles were performed. The values are given as multiples of the control group.

### 2.7. Transfection of Small Interfering RNA (siRNA)

Predesigned siRNA against human Nrf2 (Catalog Number 115762) and control scrambled siRNA (Catalog Number 4611) were purchased from Biomics (China). The human ERK1- and ERK2-specific siRNA were based on NCBI Reference Sequences (GenBank, ERK1: NM_002746.2 and ERK2: NM_002745.4). ERK1/2 siRNA and scrambled control siRNA (siControl) were purchased from Cell Signaling Technology (Beverly, MA, USA). NCM460 and HepG2-ARE-C8 cells were plated at a density of 7 × 10^5^ cells per 60 mm dish. Nrf2 siRNA (50 nM) and ERK siRNA (50 nM) were introduced into each well using Lipofectamine 2000 reagent (Invitrogen, Carlsbad, CA) according to the manufacturer's protocol. After 24 h of incubation, fresh medium was added to the dishes and the cells were cultured for another 48 h. The cells were then treated with 20 *μ*M DDO7232 for an additional 6 h and lysed for qRT-PCR.

### 2.8. Immunofluorescence of NCM460 Cells

NCM460 cells were grown on coverslips for 24 h and then treated with different concentrations of DDO7232 for 12 h. The cells were then fixed and probed with Nrf2 antibody (1 : 500). Rhodamine-labeled rabbit IgG antibody (Santa Cruz Biotechnology, Santa Cruz, CA) and the fluorochrome dye DAPI (Santa Cruz Biotechnology, Santa Cruz, CA) were used to visualize Nrf2 and cell nuclei using a laser scanning confocal microscope (Olympus FluoView FV1000, Japan) with peak excitation wavelengths of 570 nm and 340 nm.

### 2.9. ELISA

NCM460 cells were plated at a density of 7 × 10^5^ cells per 60 mm dish overnight. The cells were treated with a density gradient of DDO7232 and/or 40 mg/kg DSS for 24 h. The cell supernatant was assayed for IL-6 (EK0411, ELISA kit, Boster, China), IL-1*β* (EK0393, ELISA kit, Boster, China), and TNF-*α* (EK0527, ELISA kit, Boster, China) using double-sandwich ELISA techniques, as previously described.

### 2.10. Living Cell Microscopy

NCM460 cells were seeded in 6-well plates at 60–70% confluence per well and incubated overnight. Then, NCM460 cells were pretreated with 20 *μ*M DDO7232 for 12 h and then exposed to 40 mg/mL DSS for another 12 h. After treatment, the cell culture medium was discarded and the NCM460 cells were washed twice with 2 mL of 5% PBS. Then, cells were incubated and stained with 10 *μ*M DCFH-DA (S0033, Reactive Oxygen Species Assay Kit, Beyotime, China) in serum-free medium for 30 min at 37°C in the dark. Analysis was done with a fluorescence microscope (Olympus DP72, Japan) equipped with a U-RFL-T power supply.

### 2.11. Model of Chronic DSS-Induced Colitis

Animal studies were conducted according to protocols approved by the Institutional Animal Care and Use Committee of China Pharmaceutical University. All animals were appropriately used in a scientifically valid and ethical manner. DSS (molecular weight 36–50 kDa) (MP Biomedicals, Morgan Irvine, CA) was dissolved in distilled water to a concentration of 2%. DDO7232 was dissolved in saline and then injected intraperitoneally. Sixty female C57BL/6 mice (6–8 weeks old) were divided into six groups at random (10 mice per group): a control group (drinking water), a DSS group (2% *w*/*v*), DSS + DDO7232 groups (high: 100 mg/kg, middle: 50 mg/kg, low: 10 mg/kg, by direct intraperitoneal injection), and an olsalazine group (50 mg/kg, by direct intraperitoneal injection). Chronic colitis was induced by 4 cycles of the oral administration of 2% (*w*/*v*) dextran sodium sulfate (DSS, MP Biomedical, Solon, OH) for 7 days followed by normal drinking water for 7 days. Mice in the normal group drank normal water every day. The model group drank water mixed with DSS. The mice were monitored every other day for weight loss. At the end of the fourth cycle, the mice were sacrificed, and their colons were removed and measured for their length. The colons were fixed in 10% buffered formalin (pH 7.4) for at least 24 h for further histopathological assessment, immunohistochemical analysis, and study. Plasma was assayed for murine IL-6 (EK0411, ELISA kit, Boster, China), IL-1*β* (EK0393, ELISA kit, Boster, China), IFN-*γ* (EK0375, ELISA kit, Boster, China), MCP-1 (EK0568, ELISA kit, Boster, China), and TNF-*α* (EK0527, ELISA kit, Boster, China) using double-sandwich ELISA techniques, as previously described [28].

### 2.12. Immunohistochemistry

Colonic tissues were cut on silanized glass slides and deparaffinized three times from 10% formalin fixed, paraffin-embedded colon tissue. Staining against anti-IL-6, anti-IL-1*β* (R&D Systems, USA), and anti-Nrf2 (BS1258) (1 : 100) was performed according to the kit protocol (KeyGEN, NJ, China). Rabbit polyclonal antibody phospho-Nrf2 (Ser40) (ab76026) (1 : 100) was obtained from Abcam (Cambridge, UK). Briefly, the slides were deparaffinized. Microwaves are used for antigen retrieval. The slides were microwave-boiled twice in 10 mM sodium citrate buffer containing 0.1% Tween 20 for 10 minutes. Each section was treated with 5% hydrogen peroxide and 4% peptone casein blocking solution for 20 min to diminish nonspecific staining. The slides were incubated with primary antibodies in PBS containing 5% BSA and 10% goat serum. Biotinylated secondary anti-rabbit antibodies were added and incubated at room temperature for 40 min. Streptavidin-HRP (horseradish peroxidase) was added, and after 30 min, the sections were stained with DAB substrate. Finally, counterstaining was performed using hematoxylin [26].

### 2.13. Immunofluorescence of the Colon Tissue of C57BL/6 Mice

Colonic tissue sections (5 *μ*m) were washed in 10% PBS for 20 min, blocked with 10% goat serum, and then incubated at 4°C overnight with Nrf2 primary antibody (Abcam, Cambridge, UK) in PBS containing 1% BSA (1 : 50). After washing with PBS, the tissues were incubated at 37°C for 2 h with rhodamine-labeled rabbit IgG antibody (Santa Cruz Biotechnology, Santa Cruz, CA). Then, the slides were stained with the fluorochrome dye DAPI (Santa Cruz Biotechnology, Santa Cruz, CA). The laser scanning confocal microscope (Olympus FluoView FV1000, Japan) was used to visualize Nrf2 and the cell nuclei with peak excitation wavelengths of 570 and 340 nm [27].

### 2.14. Histopathological Examination

Specimens of the colon fixed with 10% buffered formalin were embedded in paraffin. Each section (4 *μ*m) was stained with H&E. The fixed sections were examined by light microscopy for the presence of lesions. Histological evaluation of the severity of inflammation was performed using a scoring system, by a pathologist who was blinded to the treatment.

### 2.15. Statistical Analyses

Statistical analyses were calculated using both a one-way ANOVA and the Kruskal-Wallis test for multiple comparisons. For all the tests, only a *p* value < 0.05 was considered statistically significant. All the descriptive data are reported as the mean ± SD. The GraphPad Prism software was used for the statistical analyses.

### 2.16. Synthesis

#### 2.16.1. 1H-Benzo[*d*]imidazole-5-carbonitrile (**2**)

3,4-Diaminobenzonitrile (**1**, 2.5 g; 18.75 mmol; 1.0 equivalents) was dissolved in 5 M aqueous HCl (110 mL). The mixture was heated to reflux at 105°C for 12 h. The reaction was stopped, and the mixture was cooled to room temperature, alkalized with aqueous ammonia, and stored overnight. The resulting generated solid product was collected by filtration and washed with ice water. The products were used without further purification [26]. Yield: 2.5 g (94.0%). ^1^H NMR (300 MHz, DMSO) *δ* 12.47 (s, 1H), 8.34 (s, 1H), 8.03 (s, 1H), 7.62 (d, *J* = 4.17 Hz, 1H), 7.45 (d, *J* = 8.34 Hz, 1H). MS *m*/*z*: 144.2 [M + H]^+^.

#### 2.16.2. (Z)-N′-Hydroxy-1H-benzo[*d*]imidazole-5-carboximidamide (**3**)

Compound 2 (2.5 g; 17.5 mmol; 1.0 equivalents) was dissolved in EtOH (60 mL), then K_2_CO_3_ (4.32 g; 31.5 mmol; 1.8 equivalents) and H_2_NOH•HCl (2.25 g; 32.4 mmol; 1.8 equivalents) were added; the mixture was heated to reflux for 12 h. The mixture was cooled to room temperature and diluted with diethyl ether. The product was collected by filtration, washed with water, and dried under an infrared lamp. The compound was used without further purification [26]. Yield: 1.54 g (50%). ^1^H NMR (300 MHz, DMSO) *δ* 13.10 (s, 1H), 10.86 (s, 1H), 8.50 (s, 1H), 8.32 (s, 2H), 8.03 (s, 1H), 7.74 (d, *J* = 4.23 Hz, 1H), 7.58 (d, *J* = 4.23 Hz, 1H). MS: 177.3 [M + H]^+^.

#### 2.16.3. 3-(1H-Benzo[*d*]imidazol-6-yl)-5-(4-fluorophenyl)-1,2,4-oxadiazole (DDO7232)

4-Fluorobenzoic acid (0.16 g, 1.14 mmol, 1.0 equivalents) was dissolved in DMF (3.0 mL) treated with carbonyldiimidazole (0.184 g, 1.14 mmol, 1.0 equivalents) and stirred at room temperature for 1 h. **3** (0.2 g, 1.14 mmol, 1.0 equivalents) was added to the mixed solution, and another 3 mL of DMF was added to the system. Then, the temperature was increased to 110°C, and the mixture was continuously stirred for 12–18 h. After cooling, the mixture was diluted with water and saturated aqueous NaHCO_3_ solution. The generated solid product was collected by filtration and washed with saturated aqueous NaHCO_3_ solution [26]. Yield: 26.2%. White solid; m.p. 186–187°C. ^1^H NMR (300 MHz, DMSO) *δ* 12.80 (s, 1H), 8.40 (s, 1H), 8.30–8.27 (m, 3H), 7.95 (d, *J* = 8.22 Hz, 1H), 7.78 (s, 1H), 7.55–7.49 (m, 2H). ^13^C NMR (125 MHz, DMSO) *δ* 174.128, 168.867, 164.780 (d, *J* = 251.13 Hz), 143.950, 130.581 (d, *J* = 9.25 Hz), 120.731, 120.150, 119.626, 116.609 (d, *J* = 22.34 Hz), 115.861, 114.816. IR (cm^−1^, KBr film): 3419 (−NH), 1620, 1095, 1023, 805. HRMS (ESI): calculated for C_15_H_10_FN_4_O [M + H]^+^ 281.0833, found 281.0843. HPLC (80% methanol in water): *t*_R_ = 7.43 min, 98.88%.

## 3. Results

### 3.1. DDO7232 Activated the Nrf2-ARE Pathway in NCM460 Cells

The Nrf2-ARE pathway regulates a battery of detoxification enzymes and antioxidant proteins including NQO1, HO-1, GCLM, and GCLC [10]. Therefore, the process plays an important role in the cellular defense system. NQO1 is a cytosolic flavoenzyme that exerts a chemopreventive function [29]. HO-1 is a rate-limiting enzyme in heme catabolism that functions as an endogenous defense factor [30]. Activation of Nrf2 positively regulates the transcription of NQO1 and HO-1, which are essential for reducing the risk of gastrointestinal inflammation through endogenous defense mechanisms. GCLC and GCLM play significant roles in the synthesis of glutathione (GSH), involved in cysteine ligase catalysis and rate-limiting steps [31].

**Figure 1:**
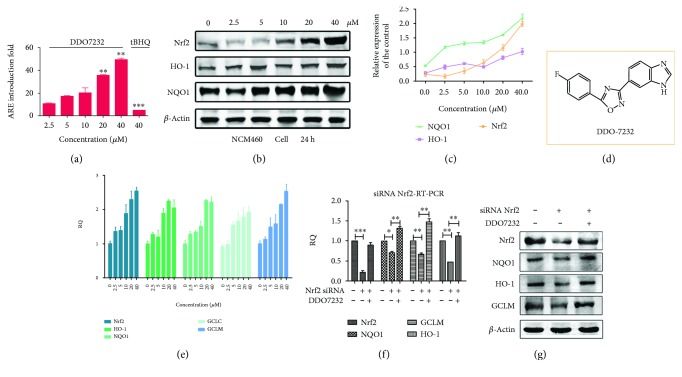
DDO7232 activated the Nrf2-ARE pathway in NCM460 cells through an Nrf2-dependent mechanism. (a) Nrf2-ARE-inducing activity of DDO7232 in HepG2-ARE-C8 cells assayed by the luciferase reporter gene assay. HepG2-ARE-C8 cells were treated with DDO7232 at gradient concentrations of 2.5, 5, 10, 20, and 40 *μ*M for 12 h. The positive control was 40 *μ*M tBHQ. The ARE-inducing activity of DDO7232 was compared with that of the DMSO control group. (b) Western blot analysis of the induction of Nrf2 and its downstream proteins after DDO7232 treatment for 24 h. *β*-Actin was used as internal reference. (c) Densitometric analysis was performed to determine the relative ratios of the protein in each fraction. The data were normalized *β*-actin expression and expressed as the mean ± SD of three individual experiments. The data were analyzed using the ImageJ 1.44p software. (d) The structure of DDO7232. (e) Quantitative real-time PCR analysis of Nrf2, HO-1, NQO1, GCLC, and GCLM in NCM460 cells. Gene transcription levels were determined after treatment with different concentrations (0, 2.5, 5, 10, 20, and 40 *μ*M) of DDO7232 for 8 h. GAPDH was used as a control for the normal transcription of these genes. (f) The mRNA transcription of Nrf2 and its downstream markers including NQO1, GCLM, and HO-1 after treatment with Nrf2 siRNA and DDO7232. NCM460 cells were treated with Nrf2 siRNA (50 nM), DDO7232 (20 *μ*M), and Nrf2 siRNA (50 nM) + DDO7232 (20 *μ*M) for 8 h. GAPDH was used as the control for the normal transcription of these genes. All gene transcription levels were determined by qRT-PCR. The values shown are mean ± SD (*n* = 3 independent assays). ^∗^ *p* < 0.05, ^∗∗^ *p* < 0.01, ^∗∗∗^ *p* < 0.001, statistically significantly different from the nontreated blank control group. (g) Western blot analysis of Nrf2 and the Nrf2-regulated proteins after exposure to Nrf2 siRNA and DDO7232. The NCM460 cells were treated with Nrf2 siRNA (50 nM) or DDO7232 (20 *μ*M) plus Nrf2 siRNA (50 nM) for 8 h. Additional NCM460 cells were treated with DMSO for use as the blank control. *β*-Actin was used as internal reference.

The ARE-luciferase reporter assay showed that DDO7232 induced ARE in a concentration-dependent manner and had higher potency than the compound tBHQ, used as a positive control. In HepG2-ARE-C8 cells, the most potent compound DDO7232 (Figure 1(d)) had an ARE-inducing activity of 45-fold at 40 *μ*M compared with tBHQ, which showed a 3-fold induced activity at 40 *μ*M (Figure 1(a)). To identify the effect of DDO7232 on the regulation of the Nrf2-ARE pathway, further experiments were performed in NCM460 cells. As shown in Figures 1(b) and 1(c), in NCM460 cells treated with DDO7232, the protein expression of Nrf2 and downstream phase II enzymes, including HO-1 and NQO1, increased in a concentration-dependent manner. Subsequently, quantitative real-time PCR (qRT-PCR) was performed to further determine the transcription of Nrf2 and antioxidant genes including GCLM, HO-1, and NQO1 to confirm these results. Nrf2, HO-1, NQO1, GCLC, and GCLM gene transcription was determined after treatment with different DDO7232 concentrations (0, 2.5, 5, 10, 20, and 40 *μ*M) for 8 h in NCM460 cells. DDO7232 significantly increased mRNA transcription in a concentration-dependent manner and exhibited a close association with protein expression (Figure 1(e)). Nrf2, HO-1, NQO1, GCLC, and GCLM gene transcription increased by 2.5-, 2.2-, 2.3-, 1.9-, and 2.7-fold, respectively, after treatment with 40 *μ*M DDO7232.

To determine whether the potency of DDO7232 was caused by Nrf2 activation, we examined the transcription of Nrf2-driven genes via knockdown of Nrf2 with siRNA. As expected, Nrf2 siRNA (50 nM) sharply decreased the gene transcription of Nrf2 compared with the control group (Figure 1(f)). When 20 *μ*M DDO7232 was added to the cells, the mRNA transcription of Nrf2 and its target proteins increased to a level comparable to that of the blank control in NCM460 cells (Figure 1(f)). Consistent with the silencing of Nrf2, markedly reduced levels of Nrf2 and its target proteins were observed in the Nrf2 siRNA-treated NCM460 cells. Cotreatment of DDO7232 and Nrf2 siRNA could reverse the downregulation of proteins (Figure 1(g)). These results demonstrated that DDO7232 activates the Nrf2-ARE pathway in an Nrf2-dependent manner.

### 3.2. DDO7232 Treatment Resulted in the Translocation of Nrf2 into the Nucleus

**Figure 2:**
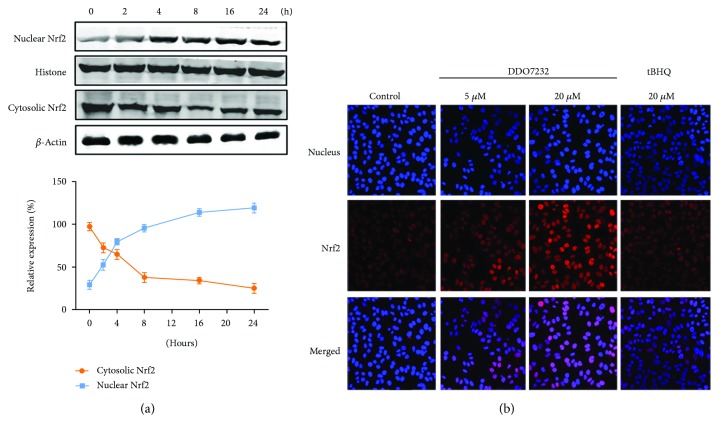
DDO7232 promoted the translocation of Nrf2 into the nucleus. (a) Nrf2 accumulated in the nucleus with the treatment of DDO7232. Nuclear and cytoplasmic extracts of NCM460 cells were obtained for Western blot analysis after treatment with DDO7232 (20 *μ*M) for different durations of time (0 to 24 h). *β*-Actin and histone were used as internal references for cytosolic and nuclear Nrf2, respectively. Densitometric analysis was used to determine the relative ratios of the Nrf2 protein in each fraction. Nuclear Nrf2 expression was normalized to histone expression, and cytosolic Nrf2 expression was normalized to *β*-actin expression. Data are expressed as the mean ± SD of three individual experiments. The data were analyzed using ImageJ 1.44p. (b) Immunofluorescence staining of Nrf2 and nuclei in NCM460 cells. The nuclei and Nrf2 were labeled with DAPI (blue) and rhodamine-labeled rabbit IgG antibody (red), respectively. The relative potency of DDO7232 at 5 *μ*M and 20 *μ*M for 12 h was assessed using the classical Nrf2 activator tBHQ as a positive control.

Nuclear translocation of Nrf2 is an indispensable process for binding to ARE and promoting transcription of the downstream cytoprotective gene. To assess the nuclear translocation of Nrf2, we measured the amount of Nrf2 in both the nucleus and cytoplasm of NCM460 cells at different time points after treatment with DDO7232 (20 *μ*M). As shown in Figure 2(a), immunoblot analysis of Nrf2 in the nucleus and cytoplasm was performed to determine whether Nrf2 accumulated in the nuclei of NCM460 cells. Nucleic Nrf2 levels increased within 2 h while cytoplasmic Nrf2 levels decreased accordingly.

To further confirm that the nuclear translocation of Nrf2 was activated by DDO7232, an immunofluorescence assay was used to visualize Nrf2 distribution. Nuclei and Nrf2 were labeled with DAPI (blue) and rhodamine-labeled rabbit IgG antibody (red), respectively. The relative potency of DDO7232 was assessed by using the Nrf2 activator, tBHQ as a control (Figure 2(b)). The fluorescence intensity in the nuclei of cells treated with DDO7232 was significantly stronger than that of the control group, indicating that the aggregation of nuclear Nrf2 is concentration dependent. The data further confirmed that DDO7232 promoted the transport of Nrf2 into the nucleus.

### 3.3. DDO7232 Activated Nrf2 by Activating the ERK1/2 Phosphorylation

**Figure 3:**
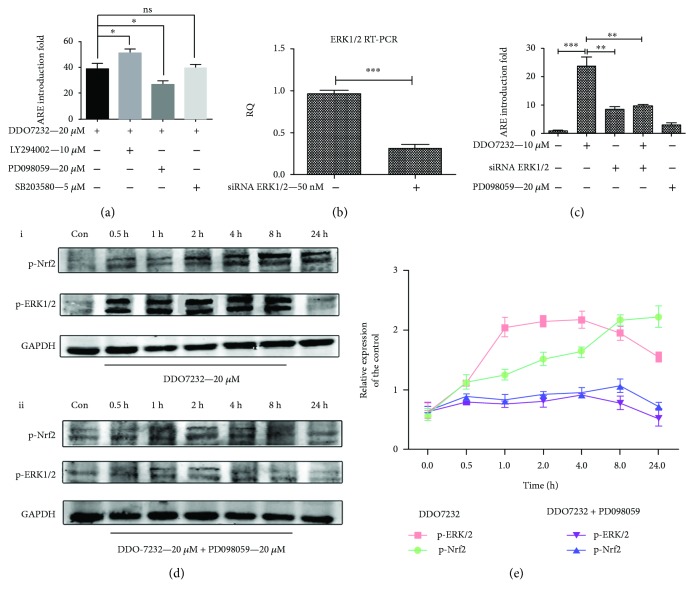
DDO7232 activated Nrf2 by inducing the ERK1/2 phosphorylation. (a) The mechanism of Nrf2-ARE activation was studied using the ARE-luciferase reporter gene assay. In HepG2-ARE-C8 cells, the ARE-inducing activity was carried out with PI3K (LY294002, 10 *μ*M), MEK1/2 (PD098059, 20 *μ*M) or P38 (SB203580, 5 *μ*M) inhibitors, and DDO7232 (20 *μ*M) for 12 h and was measured using a luciferase reporter gene assay. The activity shown is relative to the DMSO control (con). (b) HepG2-ARE-C8 cells were transiently transfected with siRNA ERK1/2 (50 nM) or control siRNA. ERK1/2 mRNA transcription was evaluated by qRT-PCR. (c) ARE-inducing activity, measured by the luciferase reporter assay, changed significantly when the HepG2-ARE-C8 cells were treated with siRNA ERK1/2 (50 nM) with or without DDO7232 (10 *μ*M). The PD098059-treated (20 *μ*M) and DMSO blank groups were used as control groups. Data are expressed as mean ± SD (*n* = 3). Differences were considered statistically significant at ^∗^ *p* < 0.05, ^∗∗^ *p* < 0.01, and ^∗∗∗^ *p* < 0.001. (d) Effects of DDO7232 (20 *μ*M) on ERK1/2 signal transduction. (i) At the indicated times after treatment with DDO7232 (20 *μ*M), NCM460 cell lysates were subjected to Western blotting of phosphorylated ERK1/2 and Nrf2. (ii) Phosphorylated ERK1/2 and Nrf2 expression as assayed by Western blot of NCM460 cells cotreated with DDO7232 and the MEK1/2 inhibitor, PD098059 (20 *μ*M), blocked activation of p-ERK1/2. Con means the DMSO control group. (e) To determine the relative ratios of the p-ERK1/2 and p-Nrf2 proteins in each fraction, densitometric analysis was performed. All the data were normalized to GAPDH expression and were expressed as the mean ± SD of three individual experiments. The data were analyzed using ImageJ 1.44p. Differences were statistically significant at ^∗^ *p* < 0.05, ^∗∗^ *p* < 0.01, and ^∗∗∗^ *p* < 0.001.

Some kinases, including mitogen-activated protein kinases (MAPK), phosphorylated p44/42 extracellular signal-regulated kinase (p-ERK), protein kinase C (PKC), and phosphoinositide 3-kinase (PI3K) participate in the phosphorylation and activation of Nrf2; however, this regulation is cell type dependent [32]. To explore which signal transduction pathway was involved in the Nrf2-ARE activation by DDO7232, we selected 3 kinase inhibitors, MEK1/2 (PD098059, 20 *μ*M), PI3K (LY294002, 10 *μ*M), and P38 (SB203580, 5 *μ*M) to cotreat with DDO7232 (20 *μ*M) in the ARE-luciferase reporter gene assay. The ARE-inducing activity decreased obviously when HepG2-ARE-C8 cells were treated with both DDO7232 (20 *μ*M) and the MEK1/2 inhibitor PD098059, which blocked the activation of p-ERK1/2 (Figure 3(a)). We presumed that DDO7232 activated the ERK1/2 signal pathway.

The direct correlation between Nrf2-ARE and ERK1/2 phosphorylation induced by DDO7232 was identified by the knockdown of ERK1/2 with siRNA (50 nM) in HepG2-ARE-C8 cells. Treating siRNA (50 nM) decreased ERK1/2 mRNA transcription to approximately 30% in HepG2-ARE-C8 cells compared with control siRNA (Figure 3(b)). The luciferase reporter gene assay was used to evaluate the ARE-inducing activity. The ARE-inducing activity of DDO7232 (10 *μ*M) reached 25-fold compared with the DMSO control group. However, the addition of siRNA ERK1/2 reduced the ARE-inducing multiple of DDO7232 to approximately 8-fold, which was similar to that of the siRNA ERK1/2 (50 nM) only group. PD098059 did not show significant ARE-inducing activity in HepG2-ARE-C8 cells, thus avoiding false positives (Figure 3(c)).

To further confirm the hypothesis, Western blot analysis was performed using proteins separated from NCM460 cells treated with or without PD098059. The results of the Western blot revealed that the phosphorylation of ERK1/2 and Nrf2 was rapid and sustained after DDO7232 treatment. The activation of ERK1/2 phosphorylation in NCM460 cells began as early as 30 min and was maximized at 8 h after exposure to DDO7232 (20 *μ*M). In addition, exposure to DDO7232 also resulted in the phosphorylation of Nrf2 Ser40 in NCM460 cells. The phosphorylation of Nrf2 in NCM460 cells began to reach equilibrium between 2 h and 8 h after treatment (Figure 3(d)). However, the cotreatment of NCM460 cells with an MEK1/2 inhibitor (PD098059, 20 *μ*M) and DDO7232 (20 *μ*M) blocked the phosphorylation of Nrf2 Ser40 and ERK1/2 (Figure 3(d)). Research confirmed that DDO7232 activated the Nrf2-ARE pathway because of the transient phosphorylation of ERK1/2 in NCM460 cells. Furthermore, the phosphorylation of Nrf2 Ser40 promoted the translocation of Nrf2 into the nucleus.

These results indicate that the Nrf2-ARE activation of DDO7232 is mediated by the ERK1/2 phosphorylation pathway. The phosphorylation of Nrf2 Ser40, induced by p-ERK, facilitated the transport of Nrf2 into the nucleus and upregulated the expression of antioxidant proteins.

### 3.4. DDO7232 Protected NCM460 Cells from DSS-Induced Injury

Oxidative stress plays a critical role in the development of chronic inflammatory diseases, including UC. The progression of UC might be mainly due to oxidative damage. As a potent Nrf2 activator, DDO7232 protected the colon against oxidative damage. DSS, a sulfated polysaccharide, is a common inflammation-inducing agent in cell and animal models of UC. To establish a model of UC, DSS-induced injury in NCM460 cells was constructed.

**Figure 4:**
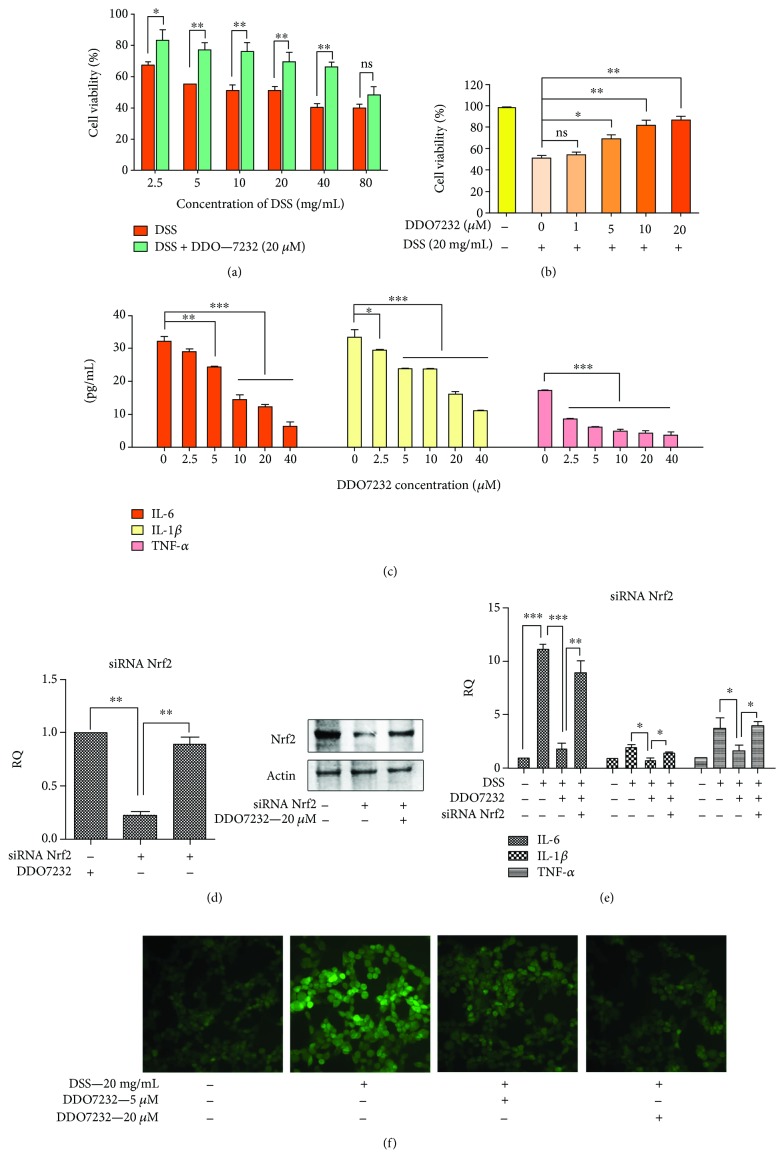
DDO7232 protected NCM460 cells from DSS-induced injury and downregulated inflammatory factors by activating Nrf2. (a) DDO7232 protected NCM460 cells from DSS-induced damage at different concentrations. The NCM460 cells were cotreated with different concentrations of DSS (from 2.5 mg/mL to 80 mg/mL) and 20 *μ*M of DDO7232 for 12 h. Cell viability was measured using the MTT assay. (b) DDO7232 protected NCM460 cells in a concentration-dependent manner to reduce DSS-induced damage. The NCM460 cells were treated with different concentrations of DDO7232 (from 1 *μ*M to 20 *μ*M) and 20 mg/mL of DSS with each group for 12 h. The cells of control group were not treated with DDO7232 or DSS. Cell viability was measured by the MTT assay. (c) DDO7232 downregulated inflammatory factors, including IL-6, IL-1*β*, and TNF-*α*, as measured in the supernatant from DSS-induced NCM460 cells. Quantification of the inflammatory factors, including IL-6, IL-1*β*, and TNF-*α*, was determined by ELISA. (d) NCM460 cells were transiently transfected with siRNA Nrf2 (50 nM) or control siRNA and then treated with DDO7232 (20 *μ*M). (d) Western blot analysis of Nrf2 after exposure to Nrf2 siRNA and DDO7232. The NCM460 cells were treated with Nrf2 siRNA (50 nM) or DDO7232 (20 *μ*M) plus Nrf2 siRNA (50 nM). Additional NCM460 cells were treated with DMSO for use as the blank control. *β*-Actin was used as internal reference. (e) IL-6, IL-1*β*, and TNF-*α* mRNA transcription after knockdown by Nrf2 siRNA (50 nM) and with DDO7232 (20 *μ*M) or without DDO7232 treatment in DSS-treated NCM460 cells. DSS, DSS + DDO7232 (20 *μ*M), and DSS + DDO7232 (20 *μ*M) + siRNA Nrf2 groups were evaluated via qRT-PCR. (f) Living cell microscopy. NCM460 cells were pretreated with 20 *μ*M or 5 *μ*M DDO7232 for 12 h and then exposed to 40 mg/mL DSS for another 12 h. After these treatments, 10 *μ*M DCFH-DA was used to stain the NCM460 cells for 20 min at 37°C, and living cell fluorescence microscopy was performed. Data are expressed as mean ± SD (*n* = 3). Differences are statistically significant at ^∗^ *p* < 0.05, ^∗∗^ *p* < 0.01, and ^∗∗∗^ *p* < 0.001.

As shown in Figure 4(a), the NCM460 cells were treated with different concentrations of DSS (from 2.5 mg/mL to 80 mg/mL). Each fraction was treated with 20 *μ*M DDO7232 for 12 h to determine the protective effects of DDO7232 by MTT assay. Cell viability decreased significantly after the treatment of various concentrations of DSS. NCM460 cell viability was reduced below 50% when the concentration of DSS was above 20 mg/mL. The results of the bioassay showed that 20 *μ*M DDO7232 protected cells against DSS-induced injury (Figure 4(a)). The protective effects of DDO7232 remained concentration dependent when treated with DSS of 20 mg/mL (Figure 4(b)). During the progression of inflammatory diseases such as UC, many overexpressed inflammatory factors may impair the normal function of the intestine when subjected to oxidative stress. Those overproduced inflammatory factors can also cause oxidative stress. We subsequently quantified several inflammatory factors in DSS-induced cell supernatants of NCM460 cells to assess the anti-inflammatory function of DDO7232. Inflammatory factors, including TNF-*α*, IL-6, and IL-1*β*, in the cell supernatant were increased in the DSS-induced NCM460 cells. When DSS-induced NCM460 cells were treated with DDO7232 (2.5 *μ*M to 40 *μ*M), all of the inflammatory factors were significantly reduced in a concentration-dependent manner (Figure 4(c)). These results show that DDO7232 reduced the amount of IL-6, TNF-*α*, and IL-1*β* in DSS-treated NCM460 cell supernatants.

To determine whether the anti-inflammatory activity of DDO7232 was due to the activation of Nrf2-ARE, Nrf2 gene interference was performed by siRNA (50 nM) and changes in the mRNA transcription of inflammatory factors were determined. The mRNA transcription of inflammatory markers, including IL-6, IL-1*β*, and TNF-*α*, in NCM460 cells treated with DSS (2%) showed a dramatic increase. The addition of DDO7232 decreased the mRNA transcription of inflammatory factors to varying degrees. However, the cotreatment of siRNA Nrf2 and DDO7232 (20 *μ*M) could not reduce the mRNA transcription of inflammatory cytokines when the mRNA and protein of Nrf2 were reduced in NCM460 cells (Figures 4(d); 4(d), D; and 4(e)). These results demonstrate that the anti-inflammatory activity of DDO7232 is closely related to the activation of Nrf2.

The intracellular ROS levels were significantly increased after DSS treatment. To investigate whether DDO7232 could counteract a DSS-induced increase in ROS in NCM460, DCFH-DA was used as a cellular ROS fluorescence indicator. As shown in Figure 4(f), ROS appeared after treatment with 20 mg/mL DSS for 12 h, and the living cell fluorescence microscopic signal produced by DCFH-DA was much stronger in the DSS-treated group than that in the control group. The pretreatment of DDO7232 (20 *μ*M or 5 *μ*M) significantly reduced the cellular DCFH-DA fluorescence intensity, which demonstrated its resistance to ROS in NCM460 cells. These data revealed that DDO7232 protected NCM460 cells from DSS-induced oxidative damage. Pretreatment with DDO7232 increased cell viability and downregulated the inflammatory cytokine content attributed to Nrf2 activation in DSS-challenged cell cultures. The cytoprotective effects of DDO7232 were partly due to the reduction of ROS induced by DSS.

### 3.5. DDO7232 Produced Cytoprotective Effects in Colon Tissues

**Figure 5:**
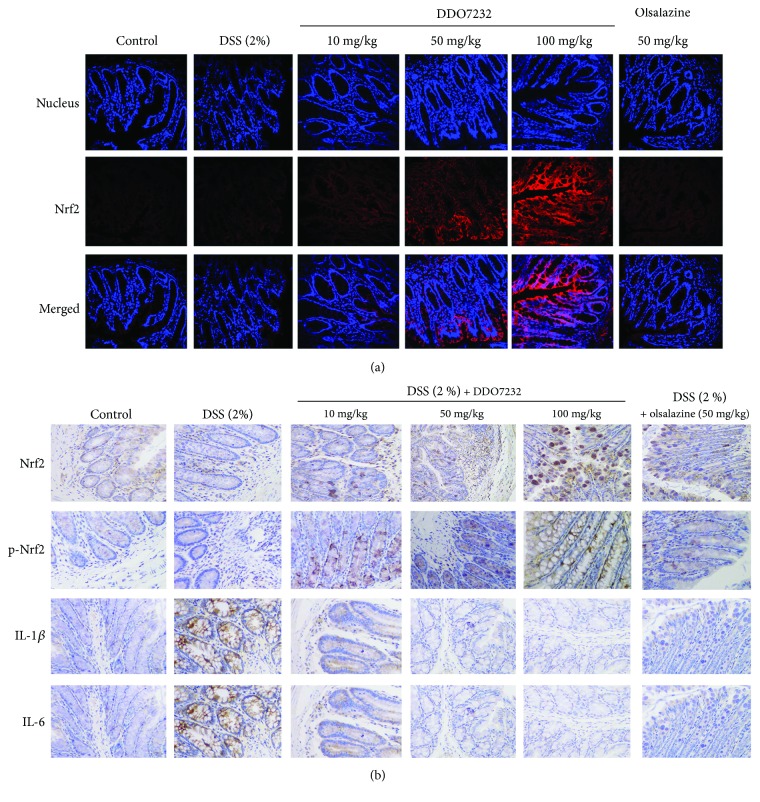
DDO7232 activated Nrf2 and reduced the DSS-induced damage caused by inflammatory factors in an UC mouse model. (a) Immunofluorescence staining for Nrf2 and cell nuclei in the colon tissues of C57BL/6 mice. The nucleus and Nrf2 were labeled with DAPI (blue) and rhodamine-labeled rabbit IgG antibody (red), respectively, and the merged image represents both. Olsalazine, a drug commonly used to treat colon colitis, was used as a positive control. Low (10 mg/kg), middle (50 mg/kg), and high (100 mg/kg) concentrations of DDO7232 were used to treat mice and compared to the blank control group that did not get DDO7232 treatment. The result shown is from one representative out of ten studied (*n* = 10). Magnification: ×200. (b) Immunohistochemical detection of Nrf2, p-Nrf2, and the inflammatory factors IL-1*β* and IL-6 in DSS-treated mice. The blank control group was treated with neither DSS nor DDO7232. The DSS-stimulated group and olsalazine-treated group were used as the model group and positive control group, respectively. Brown stains represent Nrf2, p-Nrf2, IL-6, and IL-1*β* expression, while blue represents the nucleus. The result shown is from one representative paraffinized specimen out of ten studied (*n* = 10). Magnification: ×200.

These data prove that DDO7232 activates Nrf2 *in vitro* and protects NCM460 cells against DSS-induced damage by activating Nrf2-dependent cytoprotective proteins, including HO-1 and NQO1. To further explore the potential therapeutic effects of DDO7232 on a DSS-induced UC mouse model, the immunofluorescence assay was used to detect the nuclear translocation of Nrf2 induced by DDO7232. Sixty female C57BL/6 mice (6–8 weeks old) were divided into six groups at random (ten mice per group): a control group (drinking regular water), a DSS (2% *w*/*v*) group, DSS (2% *w*/*v*) + DDO7232 groups (high dose: 100 mg/kg, middle dose: 50 mg/kg, and low dose: 10 mg/kg, by direct intraperitoneal injection), and a positive group treated with olsalazine (50 mg/kg, direct intraperitoneal injection). The detailed procedure of generating a DSS-induced UC mouse model is described in Materials and Methods. The immunofluorescence staining assay and laser confocal microscope were used to measure the expression of nucleic Nrf2 in the colon tissues of DSS-treated mice. Nrf2 was stained red, and the nuclei were stained blue. Compared with the blank control group, Nrf2 was slightly upregulated in the DSS-treated group, which may be caused by the self-preservation of C57BL/6 mice. When treated with olsalazine or DDO7232 (10 mg/kg, 50 mg/kg, and 100 mg/kg), Nrf2 was upregulated significantly in all of the groups. The DDO7232-low group showed an Nrf2 level comparable to that of the olsalazine-treated group. Nrf2 was activated by DDO7232 and translocated into the nucleus in a concentration-dependent manner (Figure 5(a)). These results demonstrate that DDO7232 promotes the efficient translocation of Nrf2 into the nucleus *in vivo*.

In addition to examining the protective effect of DDO7232 on colon tissue, we performed an immunohistochemical detection of Nrf2, p-Nrf2 (S40), IL-1*β*, and IL-6 in colonic paraffin sections of DSS-treated mice. Blue spots represent the nucleus, and brown spots represent the target proteins. As shown in Figure 5(b), the expression levels of Nrf2 and p-Nrf2 in the colon increased in a concentration-dependent manner after an intraperitoneal injection of DDO7232. In addition, the downregulation of IL-6 and IL-1*β* further confirmed the protective effect of DDO7232 in DSS-challenged mice.

### 3.6. DDO7232 Relieved DSS-Induced Damage in Mice through Downregulation of Various Inflammatory Factors

**Figure 6:**
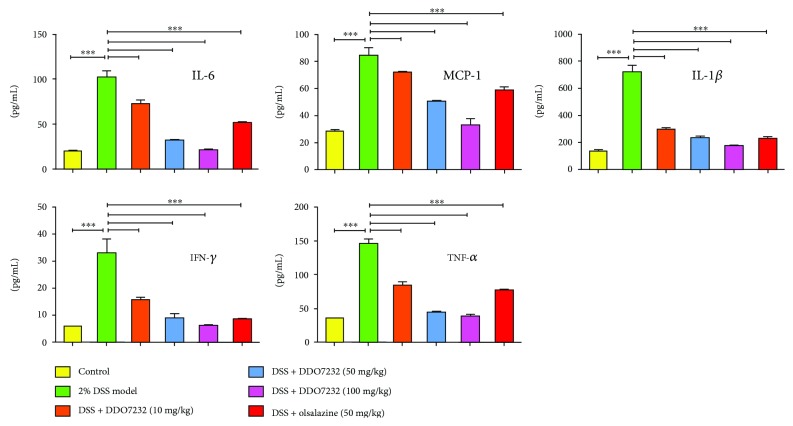
DDO7232 downregulated the inflammatory factors in the sera of mice and attenuated DSS-induced damage. The levels of various inflammatory cytokines in serum of C57BL/6 female mice were measured, including IL-6, MCP-1, IL-1*β*, IFN-*γ*, and TNF-*α*. Mice were injected intraperitoneally with different doses of DDO7232 (10 mg/kg, 50 mg/kg, and 100 mg/kg, respectively). DSS (2%) was used as a stimulant, and olsalazine (50 mg/kg) was used as a positive control. The ratios of IL-6, MCP-1, IL-1*β*, IFN-*γ*, and TNF-*α* in serum were determined by ELISA. Data are expressed as mean ± SD (*n* = 10). Differences were considered statistically significant at ^∗∗∗^ *p* < 0.001.

The production of proinflammatory cytokines, including TNF-*α*, IL-1*β*, and IL-6, is a critical indicator of inflammatory disease. The determination of inflammatory markers was an effective method to assess the effects of DDO7232 on the DSS-induced UC mouse model. The concentrations of IL-6, MCP-1, IL-1*β*, IFN-*γ*, and TNF-*α* in serum were measured by ELISA. As shown in Figure 6, all proinflammatory cytokine expression was significantly increased in the 2% DSS-treated mouse model, while the control group maintained normal expression levels. DDO7232 (10 mg/kg, 50 mg/kg, and 100 mg/kg, resp., using direct intraperitoneal injection) inhibited the expression of IL-6, MCP-1, IL-1*β*, IFN-*γ*, and TNF-*α* in the serum from DSS-challenged mice in a concentration-dependent manner. DDO7232 at high concentrations showed more potent inhibition than olsalazine. In general, DDO7232 pretreatment significantly decreased cytokine expression in DSS-challenged mice, indicating that it possessed promising anti-inflammatory activity *in vivo*. DDO7232 improved inflammatory symptoms by downregulating the mouse serum levels of IL-6, MCP-1, IL-1*β*, IFN-*γ*, and TNF-*α*.

### 3.7. DDO7232 Exhibited Potential Therapeutic Effects on DSS-Induced Chronic Ulcerative Colitis

**Figure 7:**
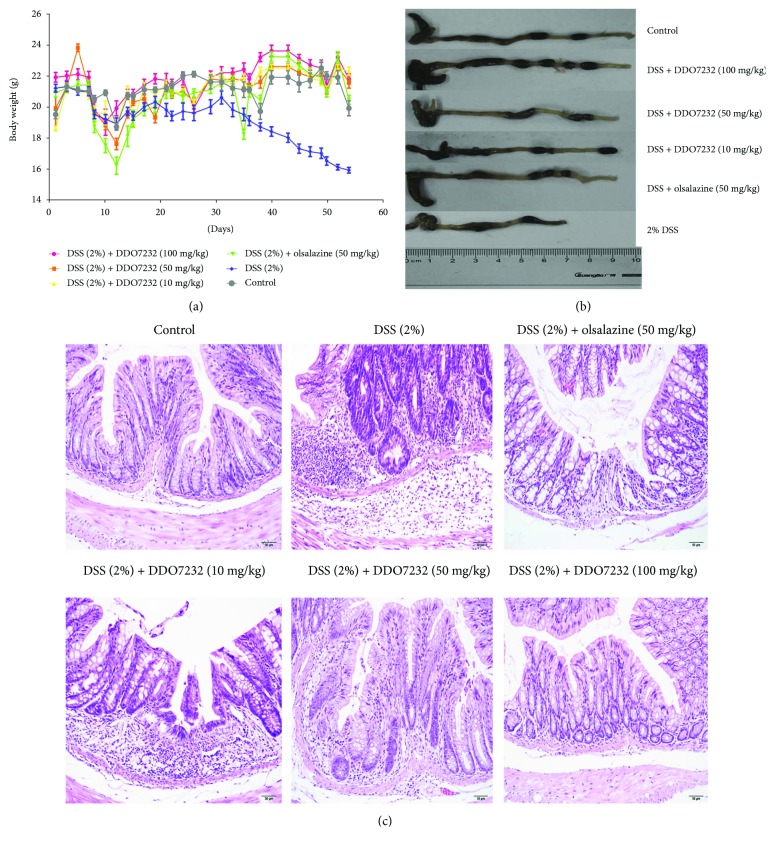
DDO7232 alleviated the pathological symptoms of DSS-treated chronic ulcerative colitis. The mouse model was divided into six groups by random assignment (ten mice per group). Mice in the control group were treated with regular drinking water. The DSS-induced group was treated with 2% *w*/*v* DSS in drinking water. DSS (treated with 2% *w*/*v* DSS in drinking water) + various concentrations of DDO7232 (10 mg/kg, 50 mg/kg, and 100 mg/kg, resp., using direct intraperitoneal injection) groups were used to determine the effects of DDO7232 on this mouse model of UC. DSS (treated with 2% *w*/*v* DSS in drinking water) + olsalazine (50 mg/kg direct intraperitoneal injection) group was set as a positive control. (a) Body weight changes of the mice in all six experimental groups (*n* = 10). (b) A comparison of colon lengths of all six experimental groups on day 55 (*n* = 10). (c) Representative histological images of distal colon sections stained with hematoxylin and eosin (H&E) to evaluate the anti-inflammatory effect of DDO7232 (*n* = 10). Magnification: ×100.

DDO7232 activates Nrf2 *in vivo* and alleviates the DSS-induced colonic inflammation. We evaluated the potential toxicity of DDO7232 by detecting the body weight of the mice every other day. Olsalazine was used as the positive control for comparison. The body weight of the mice treated with DSS was significantly reduced (Figure 7(a)), while the DDO7232 pretreatment group (both low dose and high dose groups) and the olsalazine pretreatment group body weights did not decrease significantly.

In addition, the length of the mice colons also reflected the protective effect of DDO7232. The colons of DSS-challenged mice were significantly shorter compared with those of the control group. DDO7232 and olsalazine could protect the colon from the damage of DSS. The colons of mice treated with DDO7232 and olsalazine were significantly longer than those of the DSS-challenged group. The colons of the DSS model group were approximately 5.5 cm in length and approximately 9 cm in the control group. The DDO7232 groups (low, middle, and high) and olsalazine-treated groups maintained colon lengths of approximately 7, 7.5, 9, and 8 cm, respectively.

Histological analysis of the colon crypt dysplasia was also performed using hematoxylin and eosin (H&E) staining. As shown in Figure 7(c), the DSS (2% *w*/*v*) treatment group exerted obvious inflammation (expansion of the lamina propria, disorganized distribution of the colonic mucosa, and crypt injury). The infiltration of inflammatory cells into the mucosal tissue was another typical change in ulcerative colitis. Compared with the DSS model group, treatment with DDO7232 (10 mg/kg, 50 mg/kg, and 100 mg/kg) improved the pathogenic conditions. The expansion of the lamina propria decreased. The disorganized colonic mucosa turned into well-organized colonic structures, and the inflammatory cells disappeared. All these results were DDO7232 dose dependent.

## 4. Conclusions and Discussion

Inflammatory cells play an important role in the tumor microenvironment. They accelerate the initiation, progression, and promotion of cancer. For example, chronic colitis can promote the occurrence of colon cancer [33]. Colonic mucosa is critical to the normal function of the colon and may be affected by ulcerative colitis, resulting in excessive exposure to external chemical and oxidative stress [34]. Oxidized DNA is damaged and accumulates in the inflammatory section of the colon, resulting in dysfunction or death of colonic cells [24]. The pathogenesis of UC is mainly due to the damage of the intestinal epithelium. Activation of the Nrf2-ARE pathway could provide endogenous defense systems to resist cellular oxidative stress and mitigate oxidative damage, making it a promising mechanism for inflammation therapy [35, 36]. The activation of Nrf2 downstream cytoprotective proteins, including NQO1, HO-1, and GCLM, establishes an endogenous defense system against oxidative stress. It is critical to protect the intestinal epithelium cells from physical and biochemical stimulation. In addition, activation of Nrf2 reduces cell damage and relieves pathological inflammatory responses. All these effects demonstrate that the activation of Nrf2 can balance the cytochemical microenvironment, thereby activating a series of signaling pathways against inflammation.

However, most traditional Nrf2 activators contain electrophilic groups in their chemical structures, which might bring potential side effects and toxicity [37]. Our group previously reported a series of potent Nrf2 activators with a 1,2,4-oxadiazole core. Of them, DDO7232 was the most potent one in the structure-activity relationship analysis and physical-chemical property optimization [26]. Through bioassay evaluation, we demonstrated that DDO7232 could increase the accumulation of Nrf2 in the nuclei of NCM460 cells in a concentration-dependent manner, followed by the increased transcription of downstream genes including HO-1, NQO1, GCLM, and GCLC. The siRNA Nrf2 gene interference experiments showed that the activation of downstream genes induced by DDO7232 is based on an Nrf2-ARE-dependent mechanism. Nrf2 and downstream target genes were significantly reduced with the treatment of siRNA Nrf2 alone. However, the cotreatment of DDO7232 and siRNA Nrf2 increased the mRNA transcription of Nrf2 and antioxidant genes. The results suggest that DDO7232 increased the transcription of ARE-driven genes in an Nrf2-ARE-dependent manner. DDO7232 is different from CPUY192018, which was also reported by our research group. CPUY192018 is a Keap1-Nrf2 PPI (protein-protein interaction) inhibitor, but DDO7232 is not. CPUY192018 interfered with Keap1-Nrf2 interaction directly and promoted Nrf2 transport into the nucleus. The activation of Nrf2 signaling could protect NCM460 cells and mouse colon against DSS [38].

To investigate which signaling pathway is involved in the Nrf2 activation and anti-inflammatory effect of DDO7232, we selected three upstream kinase inhibitors of MAPKs, P38, and PI3K to use in conjunction with DDO7232. The luciferase reporter gene assay showed that the ARE-inducing activity decreased significantly when cells were cotreated with the MEK1/2 inhibitor and DDO7232. Western blot and siRNA ERK1/2 interference assays confirmed that the activation of Nrf2-ARE, induced by DDO7232, was mediated by the activation of ERK1/2 phosphorylation in NCM460 colon cells. Phosphorylation of Nrf2, triggered by p-ERK1/2, facilitated the translocation of Nrf2 into the nucleus and its binding to ARE. In addition, our results demonstrated that DDO7232 protected NCM460 cells from DSS-induced oxidative damage, owing to the induction of Nrf2-targeted cytoprotective proteins. DDO7232 treatment significantly reduced DSS-induced NCM460 cell death in the cell viability assay. The siRNA Nrf2 interference assay in NCM460 cells confirmed that the decrease in inflammatory cytokines was strongly associated with Nrf2 activation.

We investigated whether DDO7232 had an *in vivo* therapeutic effect in DSS-challenged UC mice. The severe DSS-induced pathogenic symptoms were alleviated and improved by treatment with DDO7232. The immunofluorescence staining assay explored the enhanced nuclear translocation of Nrf2 in colon tissues treated with DDO7232, and the results clearly illustrated that DDO7232 not only increased the expression of Nrf2 and p-Nrf2 but also downregulated IL-1*β* and IL-6 in the colon tissues of DSS-treated mice. In addition, DDO7232 significantly reduced the levels of IL-6, MCP-1, IL-1*β*, IFN-*γ*, and TNF-*α* and other inflammation markers in mouse serum.

These data clearly indicated that DDO7232, a potent Nrf2 activator, protects NCM460 colon cells and mouse colon tissues against DSS-induced oxidative damage, and that identifies a possible mechanism for the decrease in DSS-induced inflammation. Therefore, the activation of Nrf2, induced by DDO7232, might be a beneficial option for the treatment of UC.

## Abbreviations

UC: Ulcerative colitis
DSS: Dextran sodium sulfate
IBD: Inflammatory bowel disease
CD: Crohn's disease
Keap1: Kelch-like ECH-associated protein 1
Nrf2: Nuclear factor erythroid 2 p45-related factor 2
ARE: Antioxidant responsive element
GPx: Glutathione peroxidase
GSTs: Glutathione S-transferases
NQO1: NAD(P)H/quinone oxidoreductase 1
HO-1: Heme oxygenase-1
GCLM: Glutamate-cysteine ligase modifier subunit
GCLC: Glutamate-cysteine ligase catalytic
GSH: Glutathione
qRT-PCR: Quantitative real-time PCR
NF-*κ*B: Redox-sensitive nuclear factor-B
H&E: Hematoxylin and eosin
MAPK: Mitogen-activated protein kinases
p-ERK: Phosphorylated p44/42 extracellular signal-regulated kinase
PKC: Protein kinase C
PI3K: Phosphoinositide 3-kinase.

## Supplementary Materials

Supplementary Materials Supplementary Materials: ^1^H NMR and ^13^C NMR spectra for compound DDO7232 and ^1^H NMR spectra for synthetic intermediates of compounds 2 and 3.
