# Corrigendum to “3-(1H-Benzo[*d*]imidazol-6-yl)-5-(4-fluorophenyl)-1, 2, 4-oxadiazole (DDO7232), a Novel Potent Nrf2/ARE Inducer, Ameliorates DSS-Induced Murine Colitis and Protects NCM460 Cells against Oxidative Stress via ERK1/2 Phosphorylation”

**DOI:** 10.1155/2018/8780453

**Published:** 2018-10-29

**Authors:** Li-Li Xu, Tian Liu, Lei Wang, Li Li, Yu-Feng Wu, Cui-Cui Li, Bin Di, Qi-Dong You, Zheng-Yu Jiang

**Affiliations:** ^1^State Key Laboratory of Natural Medicines and Jiangsu Key Laboratory of Drug Design and Optimization, China Pharmaceutical University, Nanjing 210009, China; ^2^Department of Medicinal Chemistry, School of Pharmacy, China Pharmaceutical University, Nanjing 210009, China; ^3^Key Laboratory on Protein Chemistry and Structural Biology and Key Laboratory of Drug Quality Control and Pharmacovigilance, Ministry of Education, China Pharmaceutical University, Nanjing 210009, China

In the article titled “3-(1H-Benzo[*d*]imidazol-6-yl)-5-(4-fluorophenyl)-1,2,4-oxadiazole (DDO7232), a Novel Potent Nrf2/ARE Inducer, Ameliorates DSS-Induced Murine Colitis and Protects NCM460 Cells against Oxidative Stress via ERK1/2 Phosphorylation” [1], there was an error in Figure 1(b) where the immunohistochemical images of IL-1*β* were erroneously imported into the immunohistochemical results of IL-6. Accordingly, the immunohistochemical pictures of IL-6 and IL-1*β* are the same set of images. The corrected version of Figure 1 is shown below.

## Figures and Tables

**Figure 1:**
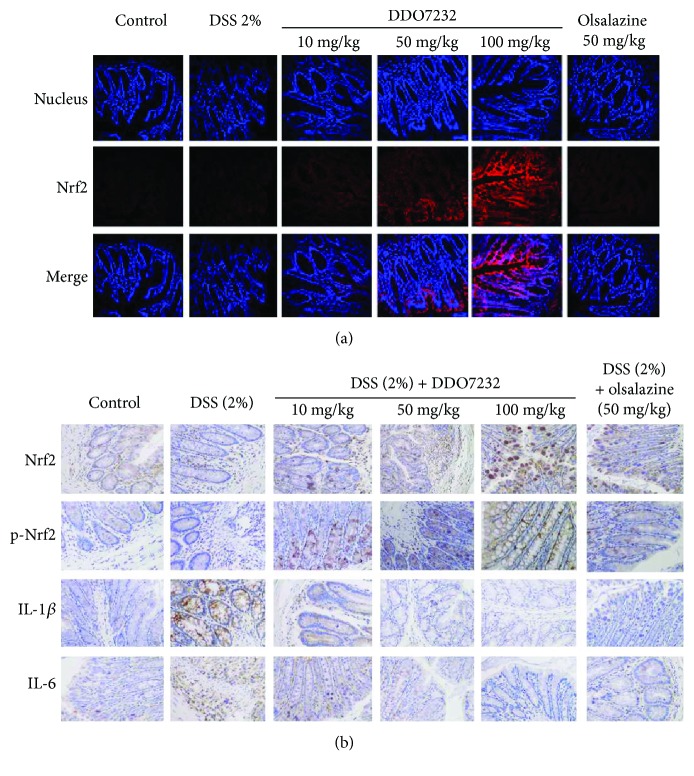
DDO7232 activated Nrf2 and reduced the DSS-induced damage caused by inflammatory factors in an UC mouse model. (a) Immunofluorescence staining for Nrf2 and cell nuclei in the colon tissues of C57BL/6 mice. The nucleus and Nrf2 were labeled with DAPI (blue) and rhodamine-labeled rabbit IgG antibody (red), respectively, and the merged image represents both. Olsalazine, a drug commonly used to treat colon colitis, was used as a positive control. Low (10 mg/kg), middle (50 mg/kg), and high (100 mg/kg) concentrations of DDO7232 were used to treat mice and compared to the blank control group that did not get DDO7232 treatment. The result shown is from one representative out of ten studied (*n* = 10). Magnification ×200. (b) Immunohistochemical detection of Nrf2, p-Nrf2, and the inflammatory factors IL-1*β* and IL-6 in DSS-treated mice. The blank control group was treated with neither DSS nor DDO7232. The DSS-stimulated group and olsalazine-treated group were used as the model group and positive control group, respectively. Brown stains represent Nrf2, p-Nrf2, IL-1*β*, and IL-6 expression while blue represents the nucleus. The result shown is from one representative paraffinized specimen out of ten studied (*n* = 10). Magnification ×200.
