# Supplementary material for: 3-(1H-Benzo[d]imidazol-6-yl)-5-(4-fluorophenyl)-1,2,4-oxadiazole (DDO7232), a Novel Potent Nrf2/ARE Inducer, Ameliorates DSS-Induced Murine Colitis and Protects NCM460 Cells against Oxidative Stress via ERK1/2 Phosphorylation
Source: Oxid Med Cell Longev. 2018 May 20;2018:3271617. doi: 10.1155/2018/3271617 (PMC5985092; doi:10.1155/2018/3271617)

Supporting Information

3-(1H-Benzo[*d*]imidazol-6-yl)-5-(4-fluorophenyl)-1,2,4-oxadiazole (DDO7232), a novel potent Nrf2/ARE inducer, ameliorates DSS-induced murine colitis and protects NCM460 cells against oxidative stress via ERK1/2 phosphorylation

Li-Li Xu, [a, b, c] Tian Liu,[a, b] Lei Wang,[a, b] Li-Li,[a, b] Yu-Feng Wu, [c] Cui-Cui Li, [c] Bin Di, [c] Qi-Dong You,[a, b*] Zheng-Yu Jiang, [a, b*]

[a] State Key Laboratory of Natural Medicines and Jiangsu Key Laboratory of Drug Design and Optimization, China Pharmaceutical University, Nanjing, 210009, China

[b] Department of Medicinal Chemistry, School of Pharmacy, China Pharmaceutical University, Nanjing, 210009, China

[c] Key Laboratory on Protein Chemistry and Structural Biology, Key Laboratory of Drug Quality Control and Pharmacovigilance, Ministry of Education, China Pharmaceutical University, Nanjing 210009, China

Table of Contents Graphic

^{1}H NMR, ^{13}C NMR spectra for representative target compounds………………S3-S4

DDO7232： ^{1}H NMR, in DMSO, 300 MHz

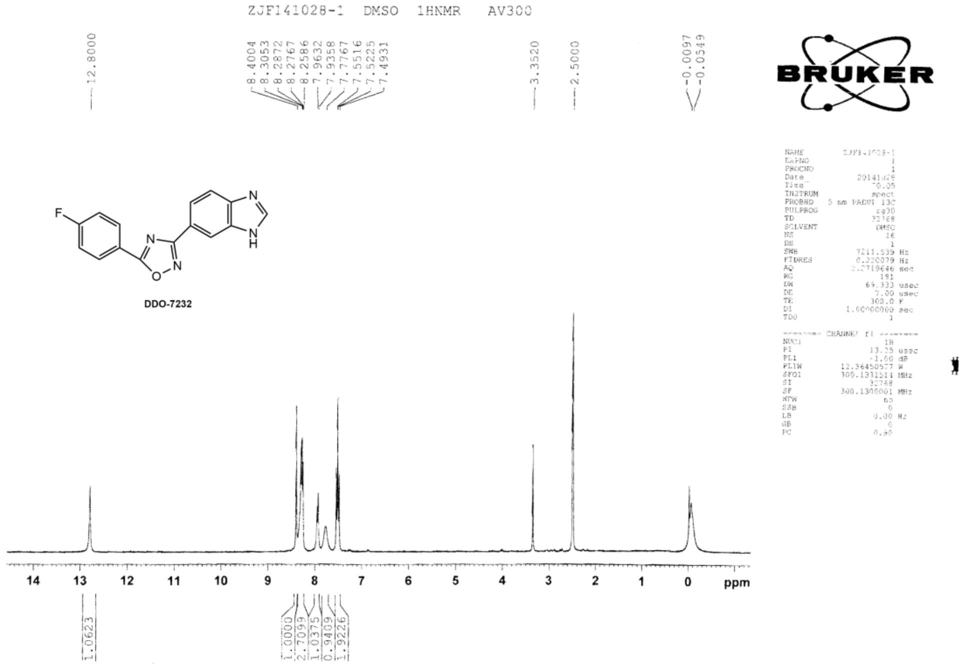

DDO7232： ^{13}C NMR, in DMSO, 75 MHz

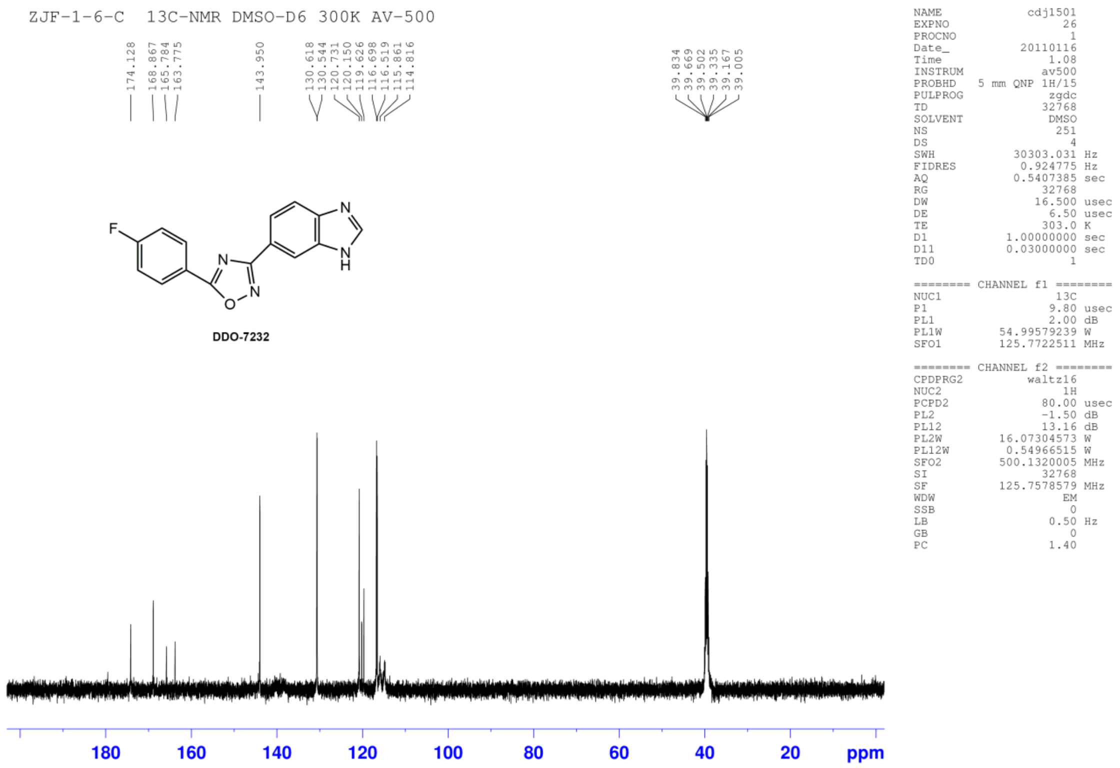

2：^{1}H NMR, in DMSO, 300 MHz

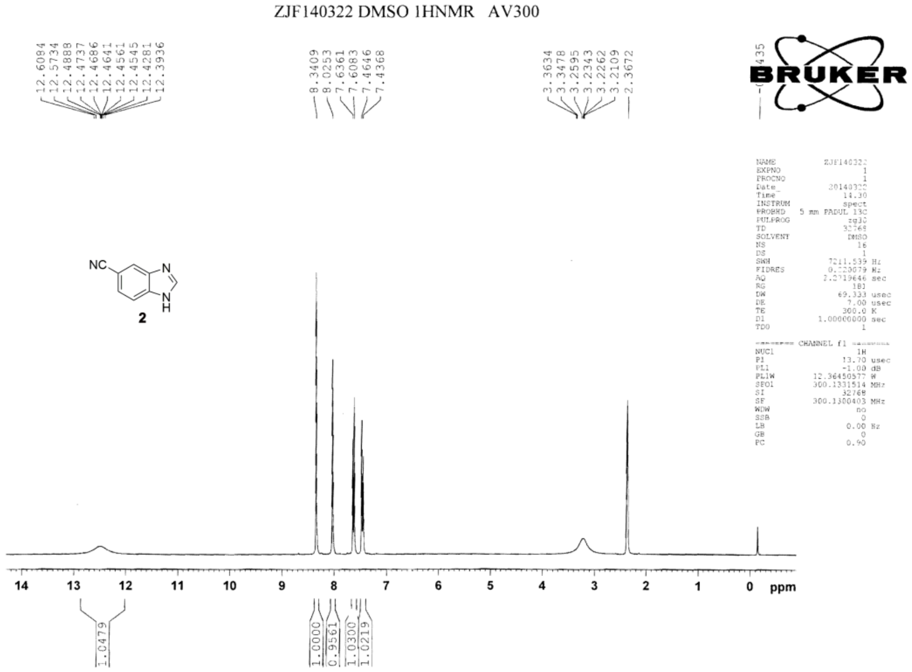

3：^{1}H NMR, in DMSO, 300 MHz

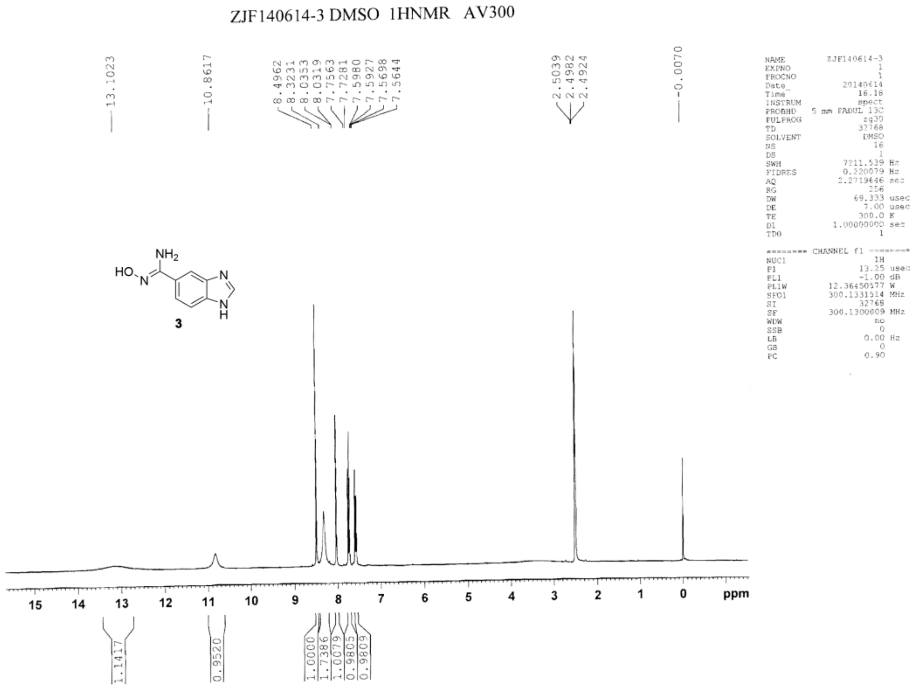

Supplement: Supplementary Materials — 1H NMR and 13C NMR spectra for compound DDO7232 and 1H NMR spectra for synthetic intermediates of compounds 2 and 3. [file 3271617.f1.pdf]
